# Multi-list interfaces for recommender systems: survey and future directions

**DOI:** 10.3389/fdata.2023.1239705

**Published:** 2023-08-10

**Authors:** Benedikt Loepp

**Affiliations:** Department of Computer Science and Applied Cognitive Science, University of Duisburg-Essen, Duisburg, Germany

**Keywords:** recommender systems, multi-list recommendation, carousels, user interfaces, user experience, choice overload, survey

## Abstract

For a long time, recommender systems presented their results in the form of simple item lists. In recent years, however, multi-list interfaces have become the de-facto standard in industry, presenting users with numerous collections of recommendations, one below the other, each containing items with common characteristics. Netflix's interface, for instance, shows movies from certain genres, new releases, and lists of curated content. Spotify recommends new songs and albums, podcasts on specific topics, and what similar users are listening to. Despite their popularity, research on these so-called “carousels” is still limited. Few authors have investigated how to simulate the user behavior and how to optimize the recommendation process accordingly. The number of studies involving users is even smaller, with sometimes conflicting results. Consequently, little is known about how to design carousel-based interfaces for achieving the best user experience. This mini review aims to organize the existing knowledge and outlines directions that may improve the multi-list presentation of recommendations in the future.

## 1. Introduction

**Figure 1:**
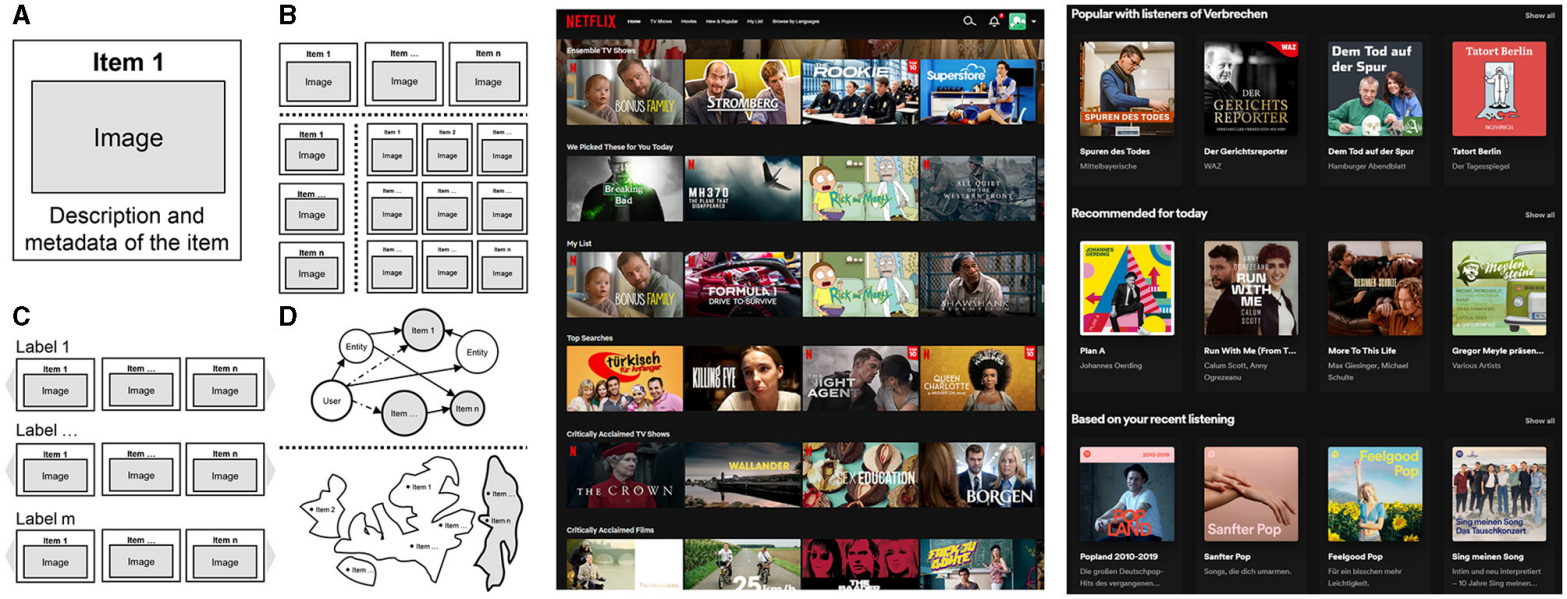
Recommender systems present their results in different ways (left), e.g., in the form of **(A)** single items or **(B)** lists of items, which may be arranged horizontally, vertically, or as a grid, but also using **(C)** carousel-based interfaces or **(D)** advanced visualization techniques such as graphs and maps. Concrete examples of carousel-based interfaces are those of Netflix (center) and Spotify (right).

Recommender systems (RS) play a vital role in a variety of domains, successfully providing users with personalized recommendations for consumer goods and entertainment media, but also for travel destinations, educational resources, people, services, and even lifestyle choices. However, the way recommendations are presented has changed significantly in recent years, especially on e-commerce and streaming platforms: While one-dimensional lists dominated for a long time, it has now become the de-facto standard to display multiple collections of recommendations. The user interfaces display these collections one below the other in a vertically scrollable list. Each row contains a number of items with a certain commonality and can be scrolled horizontally, which is why it is called a “carousel” (Bendada et al., 2020) or “shelf” (McInerney et al., 2018). Consequently, users can select items according to different contexts, rather than just from a single list optimized for a selected criterion, e.g., long-term preferences. As visible in Figure 1, Netflix shows several rows of personalized recommendations, featuring genres, popular themes, and curated content (Gomez-Uribe and Hunt, 2015). Similarly, Spotify recommends new releases, podcasts on specific topics, and songs similar users are listening to Nazari et al. (2022).

Depending on the content, different recommendation algorithms are used in the background of the carousels. Often, the systems present a corresponding label, usually as a header above the respective row. This provides a brief explanation of what is represented by the carousel, helping users identify items that match not only their general preferences, but also their current interests and situational needs. Accordingly, the carousel type can be defined based on the scheme of explanation styles proposed by Kouki et al. (2019):

- Carousels where the explanation style is *user-based* (e.g., “popular with similar viewers”) contain the results of a collaborative filtering algorithm, i.e., items well received by similar users.
- The *item-based* explanation style describes carousels that contain items similar to those that the current user has rated positively in the past (e.g., “because you watched …”).
- The *content-based* explanation style uses metadata to highlight that the items are from a certain genre, star the same cast, share similar attributes, etc. (e.g., “German pop classics”).
- The *social* explanation style (e.g., “played by friends”) refers to preferences of peers, friends, etc.
- Global *item popularity* (e.g., “top movies in Germany”) is often used for non-personalized carousels.

While all these variants are widely used in real-world applications, there is still a very limited body of literature on the presentation of recommendations in carousels. Open questions include: *which types of carousels* are preferred by which users, *how many carousels* do they want to explore, and *how many items* per carousel ensure a good decision. Moreover, while one of the main advantages of carousel-based interfaces is their ability to accommodate a variety of contexts by providing multiple sets of recommendations, it is still unclear whether this presentation format is always the most appropriate one. This is especially true when considering users with a wide range of different characteristics, aspects such as the device being used, cognitive load, and prior knowledge, as well as domain-specific requirements.

For these reasons, we aimed to organize the literature on multi-list recommender interfaces (MLRI) in this mini review. To the best of our knowledge, such a survey does not exist yet. Therefore, we systematically examined the proceedings of relevant RS and HCI conferences (e.g., RecSys, CHI, IUI, UMAP), including their workshop proceedings. We used the ACM Digital Library and Google Scholar to identify additional papers through keyword-based searches (e.g., “carousel recommendations,” “multi-list recommender interfaces”). We checked the relevance of the papers based on titles and abstracts, reviewed the relevant papers in detail, and used them for further snowballing. In Section 2, we provide an overview of the resulting set of papers. From this, in Section 3, we discuss possible directions to achieve a better understanding of carousel recommendations and to improve the design of MLRI in terms of user experience.

## 2. Multi-list recommender interfaces: an overview

In recent years, it has been gradually recognized that algorithmic accuracy and performance are not the only factors for the success of RS (Jugovac and Jannach, 2017; Loepp et al., 2019). However, the presentation of recommendations has not received the same attention as other more user-oriented aspects. Very few authors have explored alternatives to one-dimensional lists, presenting items and arranging the user interface in different ways (Lousame and Sánchez, 2009; Nanou et al., 2010; Guntuku et al., 2016; Beel and Dixon, 2021). This seems inexplicable given the potentially strong impact of the presentation format on the user experience (Knijnenburg and Willemsen, 2015). One of the most influential studies in this regard was conducted by Bollen et al. (2010). They investigated the relationship between the length of a recommendation list, the diversity of the contained items, and the occurrence of choice overload effects, and found that there is an optimal number of recommendations with respect to the balance between user satisfaction and the difficulty of making a decision. They concluded that sets of seven to ten items can be both attractive and sufficiently varied, while still being manageable for users.

The meta analysis of Scheibehenne et al. (2010) confirmed that choice overload depends on factors such as domain knowledge and decision-making strategy. However, although MLRI have become the de-facto standard in industry (cf. Section 1), most academic attempts to understand and improve the presentation of recommendations have been focused on single sets, displayed with either horizontal or vertical orientation, containing items ordered by decreasing relevance according to a selected criterion, usually long-term preferences. This also applies to the few studies in which the results were arranged as a grid. Here, the interfaces contained multiple rows, but still represented a single recommendation list, wrapped multiple times, with no option to scroll (cf. Chen and Pu, 2010; Kammerer and Gerjets, 2010).

Some of the studies on critique-based systems can be seen as exceptions, since the recommendations were displayed in groups formed on the basis of suggested critiquing options (cf. Chen and Pu, 2012a,b). However, in these cases, the purpose of item categorization was to improve the critiquing process, rather than to provide a set of diverse lists to facilitate decision making in a variety of contexts. Apart from that, the presence of categories has almost exclusively been investigated in consumer research (Knijnenburg and Willemsen, 2015). On the other hand, there exists a wide range of more advanced approaches that visualize recommendations in a more informative and appealing way than conventional lists. Numerous studies have confirmed the positive effects of graphs and maps in user-oriented dimensions such as control and transparency (He et al., 2016; Kunkel and Ziegler, 2023). However, these approaches are mostly of academic nature and too complex to be widely applied. Figure 1 provides an overview of these methods.^1^

In summary, there is a lack of research on carousel recommendations. As the next sections will show, this is especially true for questions such as those raised in Section 1, but also the impact of situational needs and individual differences. In general, personal characteristics such as expertise and decision making have not yet received much attention in RS research. The few existing studies have examined specific effects, e.g., on the preferred level of control (Jin et al., 2020), the perception of explanations (Millecamp et al., 2019), or the overall user behavior (Kleemann et al., 2021), and have always presented recommendations in a traditional way. Moreover, the literature review will show that while RS have been successful in many application scenarios (see again Section 1), research on MLRI is still limited to a few selected domains.

### 2.1. Algorithms, metrics, and models for carousel recommendations

At the same time, numerous commercial system providers have demonstrated the positive effects of carousel-based interfaces. The authors of the corresponding publications have proposed algorithmic improvements, e.g., to optimize how the collections are ordered among each other, how they are filled with items, and how labels are assigned (Wu et al., 2016, 2021; McInerney et al., 2018; Bendada et al., 2020; Lo et al., 2021). Singal et al. (2021) even investigated how to implement carousels independently of the underlying algorithms, requiring only standard user-item representations. Based on dimensionality reduction and clustering of the resulting item embeddings, this approach provides a generic way to create item collections, predict their usefulness, and find appropriate labels. Table 1 outlines the various contributions, but also shows that most of the findings stem from offline experiments and (more rarely) online A/B tests. Accordingly, metrics were used that relied purely on item clicks, largely ignoring richer behavioral data such as scrolling, responses to the mere presence of labels or items, and specific characteristics of the domain, user, or situation. Moreover, most comparisons were made against single lists, since authors (including academics) tried to optimize each collection individually (cf. Bendada et al., 2020; Jeunen and Goethals, 2021).

**Table 1:** Summary of the literature on carousel-based recommender interfaces (in chronological order).

**Paper and venue/journal**	**Topic/contribution**	**Domain**	**Carousel types**	**Experiments and datasets**
Wu et al. (2016) ACM RecSys conf.	Carousel and item ordering based on navigation signals	Video streaming	Various	Offline (private Netflix dataset)
McInerney et al. (2018) ACM RecSys conf.	Labeling and item ordering based on bandits	Video and music streaming	Various	Offline (private Spotify dataset), online A/B testing
Bendada et al. (2020) ACM RecSys conf.	Item ordering based on bandits, dataset, evaluation framework	Music streaming	Content-based (genres, location, mood)	Simulations (public Deezer dataset), online A/B testing
Felicioni et al. (2021) ACM IMX conf.	Offline evaluation of multi-list interfaces, evaluation metric	Video streaming	Various	Offline (MovieLens 10M dataset)
Ferrari Dacrema et al. (2021) ACM RecSys conf.	Carousel ordering using quantum computing	Video streaming	Various	Offline (MovieLens 10M, Netflix Prize dataset)
Jannach et al. (2021) ACM UMAP conf.	Study on user behavior with similar-item recommendation carousels	Video streaming	Various	Crowdsourced user study (some MovieLens dataset)
Jeunen and Goethals (2021) ACM RecSys conf.	Item ordering based on contextual bandits	Music streaming	Unspecified	Simulations (dataset from Bendada et al., 2020)
Lo et al. (2021) ACM RecSys conf.	Carousel ordering for similar-item recommendations	E-commerce	Various	Offline (private eBay dataset), online A/B testing
Rahdari et al. (2021) IntRS workshop	User control in multi-list interfaces	Education	Content-based (topics, keywords)	–
Singal et al. (2021) ACM RecSys conf.	Labeling, carousel and item ordering based on dim. reduction	Music streaming	Content-based	Offline (private Wynk Music dataset), online A/B testing
Starke et al. (2021) ACM RecSys conf.	Study on user behavior with similar-item recommendation carousels	Recipes	Content-based	Crowdsourced user study (crawled recipe dataset)
Wu et al. (2021) ACM WSDM conf.	Item ordering for 2-dim. product search based on log analysis	E-commerce	Unspecified	Offline (private Airbnb dataset)
Ferrari Dacrema et al. (2022) Frontiers in Big Data	Offline evaluation of multi-list interfaces, evaluation metric	Video streaming	Various	Offline (MovieLens 20M, Netflix Prize, ContentWise Impr. dataset)
Rahdari et al. (2022a) ACM RecSys conf.	User control in multi-list interfaces	Health-related documents	Content-based (topics)	–
Rahdari et al. (2022b) ACM HT conf.	Offline evaluation of multi-list interfaces, click model	Video streaming	Content-based (genres)	Simulations (MovieLens 100 K dataset)
Starke et al. (2022) IntRS workshop	Study on choice overload in carousels	Recipes	Content-based (categories)	Crowdsourced user study (crawled recipe dataset)
Starke et al. (2023) ACM TORS	Study on choice overload and personalization in carousels	Recipes	Content-based (categories)	Crowdsourced user studies (crawled recipe dataset)
Xi et al. (2023) ACM WSDM conf.	Carousel and item ordering based on attention networks	E-commerce	Unspecified	Offline (public Taobao dataset, crawled app store dataset)

However, there are some notable exceptions, where the authors have attempted to model user behavior specifically for multiple carousels. Inspired by studies on search user interfaces, Felicioni et al. (2021) assumed that users follow a “golden triangle,” i.e., their attention decreases from the top-left corner to the bottom and the right. From this, Ferrari Dacrema et al. (2022) formally defined an extension of the well-known NDCG metric, N2DCG, where the discounted cumulative gain *g* is calculated as follows:


(1) $$\begin{array}{l} \begin{array}{c} {2DCG}_{u}=\overset{V}{\underset{j=1}{\sum }}\overset{H}{\underset{k=1}{\sum }}g_{ujk}d_{jk}, \end{array} \end{array}$$

With *V* and *H* representing the number of carousels (vertical) and items (horizontal), respectively. The proposed discount function *d* takes into account both the above assumption and the number of scrolls required to reveal an item. Using the normalized version of (1) averaged over all users, the authors found that typical algorithms for implementing different carousel types perform differently when they are combined in a MLRI instead of being evaluated alone. Based on findings from three real-world datasets, they concluded that it is important to account for the availability of multiple collections when choosing an appropriate recommendation method. Consequently, selecting the right carousels becomes a very complex problem, which is why the authors recently proposed to use quantum computing to find a solution (Ferrari Dacrema et al., 2021). Aside from some of the aforementioned industry publications, few other authors have studied MLRI with such a holistic view. For instance, Xi et al. (2023) proposed an attentional re-ranking model that captures the user interaction with a whole page. They even went a step further by considering the special case of “F-shaped” pages, i.e., interleavings of vertical and horizontal collections, as well as the fact that users behave differently depending on the carousel type. The authors also reviewed the recent advances in page-level optimization, but these approaches are beyond the user-centered scope of this mini review.

Finally, Rahdari et al. (2022b) extended the cascade model, which describes user behavior in ranked search result lists (Craswell et al., 2008). Contrary to the above assumption, this resulted in a carousel click model that simulates user interaction under the premise that before users begin to examine the items, they explore vertically until they find a collection with a label that catches their attention. For the corresponding experiment, the authors chose labels based on genre information from the *MovieLens* dataset, i.e., only simple content-based explanations. The main finding was that the simulated users were more efficient than when scanning one-dimensional lists. Rahdari et al. (2021) also explored how to improve interactive control in MLRI by allowing users to fine-tune the importance of the topics represented by individual carousels. In a recent publication, they further demonstrated the successful use of carousels in a more practical domain, i.e., medical advice (Rahdari et al., 2022a). Without user studies, however, the empirical basis for the design of MLRI still remains weak, especially in light of other domains, where the user experience may be different depending on, e.g., item complexity and user familiarity.

### 2.2. User experiments on carousel recommendations

Among the publications listed in Table 1, only a few report a user experiment. Jannach et al. (2021) conducted a large online study to investigate the impact of different design alternatives (*N* = 775). Their exploratory study provided initial insights into the usage and assessment of carousels in the context of similar-item recommendations: Participants were slower in their decision making when they were confronted with multiple lists, but explored longer before settling on a movie. Compared to a grid without labels, the grouped organization also increased the perceived diversity and novelty of the recommended items, remarkably even with labels that did not have a meaning. With respect to labeling, the study also showed that user- and item-based carousels were preferred over references to, e.g., movie genre, director, or release date. Finally, it is worth noting that removing duplicate items did not make a difference.

Starke et al. (2022) compared a carousel-based recipe recommendation interface with a conventional vertical list and a grid (*N* = 150). Although the carousels had descriptive labels, they found no positive effects on choice satisfaction or difficulty compared to the grid, where the rows had no explanations and could not be scrolled horizontally. The authors noted several reasons for this finding, but it could also have been the result of the very specific task (“find the most suitable vegetarian recipe”) combined with the fact that the dataset consisted only of vegetarian dishes and the labels were not very distinctive (e.g., “vegetarian recipes,” “salad recipes”). However, compared to the list, both the grid and the carousels were perceived as easier to use, although it was more difficult to choose an item. Other aspects related to user experience, such as carousel length or individual decision-making traits, were not taken into account.

In another study (*N* = 366), however, Starke et al. (2021) examined the effects of personal characteristics and explanation styles. They found that carousels had a positive effect on choice satisfaction and perceived diversity. On the other hand, but consistent with the literature (Iyengar and Lepper, 2000), participants needed more time to make a decision than with a conventional list. While cooking experience was positively correlated with comprehensibility and satisfaction, there were no interaction effects, i.e., the MLRI had no general advantage. Moreover, no differences were found when comparing carousels with and without explanations. Apparently, labels such as “similar recipes that contain fewer calories” neither made the decision easier nor led to greater satisfaction with the chosen item. Since this contradicted earlier findings on grouped interfaces (see above), the authors concluded that it still needs to be investigated whether item details, images, or descriptive texts are more critical for making decisions than carousel labels. However, it is also important to note that the study was limited to similar-item recommendations. Given the very specific domain and the interface, which was quite different from real-world systems (few recommendations, no personalization), it is therefore difficult to generalize the results.

Only recently, Starke et al. (2023) conducted another study in their series of experiments on using carousels to promote healthy food choices (*N* = 164). Again, they compared a single- with a multi-list format, but also varied the personalization of the labels. While the results were consistent in terms of diversity and comprehensibility, participants were less satisfied with their choices in the multi-list condition, contrary to the findings above. Moreover, the previously observed differences in choice difficulty were not present. Regarding personalization, the authors found that labels without a focus on nutrition were preferred, e.g., “these recipes [match] your low level of cooking experience.” The personalization also led to unhealthier recipe choices, possibly because participants developed negative feelings when the explanations were explicit about the relationship between personal characteristics and nutritional value. However, as acknowledged by the authors, some of the findings, including those related to the influence of health consciousness and domain knowledge, require further confirmation, especially since it was not possible to fit a structural equation model to analyze mediating effects in more depth. Besides, the relatively small recommendation sets and the fact that the crowdworkers participating in the study probably did not consume the chosen recipes may have compromised the ecological validity.

## 3. Summary and future research directions

The literature review has shown that in MLRI, the effects of personal characteristics and situational needs on aspects such as cognitive load and user behavior have not yet been studied to the same extent as in conventional lists. As is common in the RS field, many algorithmic advances have been proposed (see Section 2.1), but with a focus on item click data, objective metrics, and offline evaluation, partially based on assumptions that have not been validated in user experiments. In fact, there are only a few user studies available (see Section 2.2), and they do not paint a consistent picture. Instead, they have explored general design considerations in a few selected domains and with rather artificial systems^2^, focusing on comparisons against conventional lists and grids, but leaving carousel-specific questions such as those raised in Section 1 and the corresponding user decision processes largely untouched.

This lack of empirical, user-centered research is particularly problematic because carousel recommendations are often personalized, but without considering the individual user experience, which also depends on aspects such as the number of carousels, their type, size, and order, as well as the selection and ranking of the items contained. Thus, we end this survey with a discussion of the directions in which future research should proceed:

- **Interface layout, carousel design, and labeling:** Specific interface aspects, such as the number and order of the collections displayed, their visible length, or the number of items they contain, still need to be investigated in user experiments with respect to their effects on decision making and the occurrence of choice overload effects. With a better understanding of the interface layout and the design of individual carousels, it will then be possible to dynamically adjust these parameters, which in turn is a prerequisite for not only offering personalized collections, but also adapting the entire interface to the current context, i.e., improving user experience of carousel recommendations at the page level. In this regard, it may also be worth exploring how to better direct the user's attention to specific carousels, e.g., by visually highlighting relevant carousel types or adding more informative labels. Decoupled from the simple explanation styles that are currently used, but tailored more strongly to the domain, user, and current situation, this could be another important step toward reducing choice difficulty, even in an actionable way, e.g., by providing additional explanations on demand.
- **Personal characteristics and situational needs:** Any attempt to balance choice overload and the desire to explore across and within carousels will likely result in a different user experience depending on personal characteristics and situational needs: In some domains, some users may prefer a large set of diverse alternatives, while for others or in other situations, the presence of dozens of item collections may be overwhelming, possibly even leading to choice deferral (cf. Chernev et al., 2015). Thus, similar to research on one-dimensional lists, aspects such as maximization tendency (Parker et al., 2007) and decision style (Hamilton et al., 2016), but also aspects of the current context, e.g., cognitive load and domain knowledge, still need to be investigated with respect to their impact on exploration behavior (e.g., vertical and horizontal navigation depth) and selection of items from individual collections. With additional user studies, it will then be possible to draw a more consistent picture of the usage and assessment of MLRI than previous work, paving the way for more accurate user modeling, subsequent adaptation of the presentation, and ultimately better user experience.
- **Domains and datasets:** While carousels are used in almost all types of real-world applications, industry publications have only addressed e-commerce and music or video streaming. Thus, user experiments in these domains are rare, so that little is known beyond what can be inferred from clicks on the items contained in the collections. Simultaneously, few academics have conducted more user-oriented research, primarily in more serious domains, e.g., food and health (cf. Section 2.2). Given the other limitations mentioned above, it is therefore difficult to generalize their findings and to disentangle the effects of the specific use case from the influence of individual differences and aspects such as carousel type, number, and length. Moreover, existing studies did not consider item consumption, although it can strongly affect the assessment of recommendations, even in simpler domains (cf. Loepp et al., 2018). Therefore, future studies should be conducted in a wider range of domains and complemented by offline experiments and simulations. This, in turn, will require the creation of datasets that include other types of user feedback than item-related preference signals, i.e., behavioral data such as scrolling, data on the visibility and perception of carousels and items, etc.
- **Environments, devices, and modalities:** To date, MLRI have only been studied in typical web contexts, i.e., study participants had to interact with (artificial, sometimes static) web applications using a laptop or desktop computer. In practice, however, carousels are much more common on mobile devices or TVs, requiring interaction by touch or remote control. Accordingly, there is a need for studies in more naturalistic environments to better understand user behavior and decision making in relation to available carousels and interface layout. This is particularly true because the ability to satisfy diverse contexts is likely to play a much larger role in real-world applications than in the crowdsourced experiments conducted so far, where the task was predefined and focused on a single specific goal. Moreover, such studies will be useful for investigating the implementation of more explanatory labels (see above), especially if they incorporate eye-tracking analyses. Then, with richer data than item clicks, it will also be possible to validate (or reject) existing assumptions about user behavior and to obtain more comprehensive models of user interaction, which can subsequently be used to further improve the user experience of MLRI.

## Author contributions

The author confirms being the sole contributor of this work and has approved it for publication.

## Footnotes

^1^Note that there are slight differences in the definition of single- and multi-list user interfaces (cf. Jannach et al., 2021; Starke et al., 2022).

^2^Recommendation sets were often small, horizontal scrolling was not always possible, and the focus was often on similar-item recommendations, i.e., a reference item was visible at the top, whereas most real-world applications present self-contained carousels directly on the landing page.
